# A Case of Hepatitis B Virus Reactivation Triggered by Acute Epstein-Barr Virus Infection

**DOI:** 10.7759/cureus.18676

**Published:** 2021-10-11

**Authors:** Azizullah Beran, Mohammed Mhanna, Hossein Haghbin, Jack W Sample, Jordan Burlen

**Affiliations:** 1 Internal Medicine, University of Toledo, Toledo, USA; 2 Gastroenterology and Hepatology, University of Toledo, Toledo, USA

**Keywords:** hepatitis b infection, hbv reactivation, ebstein bar virus, hepatitis virus

## Abstract

Reactivation ofHepatitis B virus (HBV) is not an uncommon condition. It is known to occur with immunosuppressive therapy. There are several viral infections that can trigger HBV reactivation, such as human immunodeficiency virus (HIV) infection. However, there is no reported case of HBV reactivation triggered by Epstein-Barr virus (EBV) infection in the literature. To our knowledge, we report the first case of reactivation of HBV secondary to acute Epstein-Barr virus (EBV) infection in the literature.

A 47-year-old Caucasian male with a remote history of resolved acute Hepatitis B virus infection presented to our hospital with severe acute hepatitis, which manifested as epigastric pain, jaundice, dark urine, light-colored stools, hyperbilirubinemia, and transaminitis in the 1000s. Ultimately, the patient was diagnosed with reactivation of HBV triggered by acute EBV infection. After several days of supportive treatment, his hepatic function normalized. He was discharged with a scheduled follow-up at a hepatology clinic. In conclusion, EBV infection should be suspected as a trigger in cases with HBV reactivation, particularly when common etiologies are excluded.

## Introduction

Hepatitis B virus (HBV) reactivation is not an uncommon condition [1]. It is estimated that 248 million persons have chronic HBV infection in the world [1]. HBV reactivation is also a clinically significant cause of fulminant hepatic failure in the United States (US) [2]. The reactivation of HBV is usually provoked by B-cell depleting immunosuppressive medications such as rituximab [2]. Some factors, including coinfection with the hepatitis C virus (HCV) and human immunodeficiency virus (HIV), increase the risk of reactivation [2]. The prevalence of infection with Epstein-Barr virus (EBV) is estimated to be more than 90% of all adults in the world [3].

A case of acute HBV and EBV coinfection has previously been reported in the literature [4]. This reported case of dual infection with HBV and EBV presented with severe jaundice and coagulopathy [4]. However, to the best of our knowledge, our patient is the first case of HBV reactivation triggered by acute EBV infection reported in the literature [5]. We emphasize the importance of expanding the differentials of HBV reactivation to include less common hepatotropic agents, such as EBV. 

## Case presentation

A 47-year-old Caucasian man with a remote history of resolved acute HBV infection 30 years ago (with complete HBs Ag seroconversion) presented with a history of progressively worsening abdominal pain and icterus for two weeks. The patient reported that the pain started in the lower abdomen and moved to the epigastric and left lower quadrant. The patient also noticed the darkening of his urine and light-colored stools. He denied acetaminophen, illicit drug, herbal products, or heavy alcohol use. The patient had a history of resolved acute hepatitis B infection with jaundice and hospitalization when he was 17-years-old.

Physical examination showed jaundice and tenderness of the right upper quadrant of the abdomen. Laboratory tests on admission showed aspartate aminotransferase (AST) 1809 mg/dL, alanine aminotransferase (ALT) 2497 mg/dL, alkaline phosphatase 243 mg/dL, and total bilirubin 7.1 mg/dL with a direct bilirubin 4.4 mg/dL. His basic metabolic panel (BMP) and complete blood count (CBC) were otherwise mostly unremarkable. The serologic tests for hepatitis E virus (HEV), HIV, HCV, hepatitis D virus (HDV), herpes simplex virus (HSV), and cytomegalovirus (CMV) infections were negative. The autoimmune hepatitis panel was negative, as well. On further evaluation, the patient was diagnosed with HBV reactivation since HBs Ag, HBe Ag, and HBc IgM Ab were positive. In addition, he was also diagnosed with acute EBV infection since EBV nuclear Ag and EBV viral capsid antigen (VCA) IgM were positive. The patient had unremarkable liver enzymes and negative HBc IgM Ab in 2018, indicating he had resolved infection. Abdominal ultrasound with duplex was ordered that showed echogenic liver with a patent portal vein, hepatic vein, and hepatic artery. The serological tests and the trend of liver function tests for the patient during hospitalization are shown in Table 1 and Table 2 respectively. We diagnosed the patient with HBV reactivation secondary to acute EBV infection. The patient did not have hepatic encephalopathy, and his international normalized ratio (INR) did not increase more than 1.3. He was managed supportively. During his hospital course, his abdominal pain resolved. His transaminitis also improved, and he was discharged home with planned outpatient treatment. On further follow-up, his jaundice and dark urine resolved as well.

**Table 1:** Serological test results for viral causes of hepatitis in the hospital course. EBV VCA IgM - EBV viral capsid antigen (VCA) IgM; EBV VCA IgG - EBV viral capsid antigen (VCA) IgG; HBcAb - hepatitis B core antibody; HBeAb - antibody to hepatitis B envelope antigen; HBeAg - hepatitis B envelope antigen; HBsAb - antibody to hepatitis B surface antigen; HBsAg - hepatitis B surface antigen; HSV PCR - herpes simplex virus by polymerase chain reaction.

Test	Results
EBV VCA IgM	Positive
EBV VCA IgG	Positive
EBV nuclear Ag	Positive
HSV PCR	Negative
CMV IgM	Negative
Hepatitis e IgG	Negative
Hepatitis D total Ab	Negative
Hepatitis Anti HBe Ab	Negative
Hepatitis Bs Antigen	Positive
HBc IgM Ab	Positive
HBc IgG	Negative
HBe Ag	Positive

**Table 2:** Liver function test trend in the hospital course. AST - Aspartate aminotransferase; ALT - Alanine aminotransferase.

Tests	On admission	On discharge
AST	1809 mg/dL	1015 mg/dL
ALT	2497 mg/dL	1851 mg/dL
Total bilirubin	7.1 mg/dL	4.0 mg/dL
Direct bilirubin	4.4 mg/dL	-
Alkaline phosphatase	243 mg/dL	242 mg/dL

## Discussion

HBV reactivation risk factors are categorized into three groups: host factors, virologic factors, and immunosuppression factors [6]. Male gender is a host risk factor for reactivation that was present in our patient. Regarding virologic risk factors, the non-A genotype of HBV is more prone to be reactivated [7]. HCV and HIV coinfection present another virologic risk for reactivation. Treatment of the above pathogens without targeting HBV portends the risk of HBV reactivation [8].

Another important factor causing severe reactivation of HBV is B-cell- depleting biologics such as rituximab. This medication has an increased risk of HBV reactivation if the patient already has a B-cell immunity defect, as in lymphomas [9]. Recent research has shown that B-cell function is important in both resolutions of the acute phase and suppression of reactivation of HBV [10, 11].

EBV, also called human herpesvirus 4, is an infection to which 95% of the adult population is exposed [12]. Like other herpesviridae, the virus has the capability to become latent or cause lytic replication. The virus initially infects the nasopharyngeal epithelium CD21 receptor through intimate contact. It subsequently infects B-cells through the same receptor [12]. The best-known clinical manifestation of EBV is acute infectious mononucleosis (IM) [12]. Nausea, vomiting, and anorexia are manifestations of IM hepatitis, which is usually mild [12]. EBV can cause B-cell immune system and liver pathology [12]. We speculate that B-cell immunosuppression and hepatotoxicity by EBV led to HBV reactivation in our patient.

On reviewing the literature, there was no reported case of reactivation of HBV triggered by EBV. Coinfection of HBV and EBV was reported by Rao et al. which EBV coinfection led to a severe case of hepatitis [4]. Other pathogens such as HCV and HIV have been shown to increase the chance of HBV reactivation [6]. When treating these viruses, tailoring treatment per algorithm is recommended to address concurrent HBV. Considering the high incidence of EBV, we recommend that guidelines should be revised to factor in the risk of HBV reactivation in IM patients at high risk for HBV.

To our knowledge, this is the first case of HBV reactivation triggered by acute EBV infection reported in the literature. HBV reactivation usually occurs in B-cell-depleting chemotherapy; however, EBV by itself caused reactivation in our case. This patient’s HBV reactivation due to EBV infection was relatively mild. EBV infection should be considered in the differential diagnosis of cases with HBV reactivation, particularly when common etiologies are excluded.

## Conclusions

To our knowledge, this is the first case of HBV reactivation triggered by acute EBV infection reported in the literature. HBV reactivation usually occurs in B-cell-depleting chemotherapy; however, EBV by itself caused reactivation in our case. This patient’s HBV reactivation due to EBV infection was relatively mild. EBV infection should be considered in the differential diagnosis of cases with HBV reactivation, particularly when common etiologies are excluded.
